# Highly Sensitive Pressure Sensor Based on Elastic Conductive Microspheres

**DOI:** 10.3390/s24051640

**Published:** 2024-03-02

**Authors:** Zhangling Li, Tong Guan, Wuxu Zhang, Jinyun Liu, Ziyin Xiang, Zhiyi Gao, Jing He, Jun Ding, Baoru Bian, Xiaohui Yi, Yuanzhao Wu, Yiwei Liu, Jie Shang, Runwei Li

**Affiliations:** 1 CAS Key Laboratory of Magnetic Materials and Devices, Ningbo Institute of Materials Technology and Engineering, Chinese Academy of Sciences, Ningbo 315201, China; lizhangling@nimte.ac.cn (Z.L.); zhangwuxu@nimte.ac.cn (W.Z.); liujinyun@nimte.ac.cn (J.L.); xiangziyin@nimte.ac.cn (Z.X.); gaozhiyi@nimte.ac.cn (Z.G.); yixiaohui@nimte.ac.cn (X.Y.); wuyz@nimte.ac.cn (Y.W.); liuyw@nimte.ac.cn (Y.L.); 2 Zhejiang Province Key Laboratory of Magnetic Materials and Application Technology, Ningbo Institute of Materials Technology and Engineering, Chinese Academy of Sciences, Ningbo 315201, China; 3 College of Materials Science and Opto-Electronic Technology, University of Chinese Academy of Sciences, Beijing 100049, China; 4 School of Materials Science and Engineering, Shanghai University, Shanghai 200072, China; guantong@nimte.ac.cn; 5 School of Software and Electrical Engineering, Swinburne University of Technology, Melbourne 3122, Australia; lotusjing1@126.com; 6 Department of Materials Science and Engineering, National University of Singapore, Singapore 119260, Singapore; msedingj@126.com

**Keywords:** elastic pressure sensor, PDMS conductive microspheres, MXene–SWCNT, electrostatic self-assembly, 3D printing

## Abstract

Elastic pressure sensors play a crucial role in the digital economy, such as in health care systems and human–machine interfacing. However, the low sensitivity of these sensors restricts their further development and wider application prospects. This issue can be resolved by introducing microstructures in flexible pressure-sensitive materials as a common method to improve their sensitivity. However, complex processes limit such strategies. Herein, a cost-effective and simple process was developed for manufacturing surface microstructures of flexible pressure-sensitive films. The strategy involved the combination of MXene–single-walled carbon nanotubes (SWCNT) with mass-produced Polydimethylsiloxane (PDMS) microspheres to form advanced microstructures. Next, the conductive silica gel films with pitted microstructures were obtained through a 3D-printed mold as flexible electrodes, and assembled into flexible resistive pressure sensors. The sensor exhibited a sensitivity reaching 2.6 kPa^−1^ with a short response time of 56 ms and a detection limit of 5.1 Pa. The sensor also displayed good cyclic stability and time stability, offering promising features for human health monitoring applications.

## 1. Introduction

In recent years, flexible pressure sensors have shown promising application prospects in cutting-edge fields such as wearable electronics [1,2,3], intelligent robots [4,5,6,7], health monitoring [8,9,10,11], biomimetic electronic skin [12,13,14], and human–computer interaction [15,16,17] owing to their abilities to stretch, compress, bend, and seamlessly adhere to non-flat surfaces. Tremendous research effects have been devoted to improving the key performance of the flexible pressure sensor in terms of sensitivity, response time, and detection range [18,19,20,21,22]. For instance, resistive flexible pressure sensors are advantageous because of their high flexibility, sensitivity, simple operation, reliability, and large pressure detection range, and can match integrated technologies [23]. These features are extremely important in sensing applications of tactile perception, human–computer interaction, medical and health care, etc. However, some problems, such as complicated production processes and difficulty in achieving both high softness and high sensitivity, are still encountered. Therefore, tremendous research and development has been carried out on the optimization of flexible substrate materials to improve the performance of pressure sensors [24,25], such as through the use of conductive sensitive materials [26,27,28], design of sensitive units [29,30], and manufacturing of array structures [31,32,33,34,35]. In this quest, the optimization of flexible pressure-sensitive materials and the introduction of microstructural design have been typically conducted to improve sensor performance.

Currently, the methods commonly used to introduce microstructures into resistive pressure sensors include the construction of flexible substrate microstructures and the building of flexible pressure-sensitive material microstructures. The former strategy mainly involves the template method. For instance, Xuan et al. [36] constructed a micro-ridge and semi-dome structure on the flexible substrate surfaces through laser etching. The resulting sensors with a semi-dome structure exhibited a sensitivity of 1.82 kPa^−1^, which was found to be about 17 times higher than those of the sensors based on long micro-ridge structures. However, the processes involved in this type of technology are usually complex, prone to environmental problems, and require a very high level of craftsmanship. 

Ginkgo biloba leaves [37], lotus leaves [38], reed leaves [39], and other biological templates with abundant surface structures have also been explored as flexible substrate microstructures. For example, Wang et al. [37] used the surface of Ginkgo biloba leaf as a template and employed magnetron sputtering technology to obtain a flexible electrode with a micro-raised structure. The resistive flexible pressure sensor based on this structure exhibited an excellent sensitivity of up to 5.9 kPa^−1^, and a detection range of 0–15 kPa. However, despite the low cost and environmental friendliness, the microstructures of biological templates are difficult to control, and significant differences occur between batches of templates, which affects the stability of sensors. Accordingly, various conductive functional fillers, such as carbon materials (carbon black, reduced graphene oxide, carbon nanotubes (CNTs)) [40], metal-based materials (gold and silver nanoparticles/wires) [41], functional polymers (polypyrrole, polyaniline, etc.) [42,43], and emerging functional materials such as MXene [44] have been explored as flexible pressure-sensitive materials. However, much attention is also being paid to the exploration of the microstructure of flexible pressure-sensitive materials mainly inside the functional layer; in contrast, the surface microstructure of flexible pressure-sensitive materials has rarely been studied to date. Therefore, the development of a low-cost and simple method to construct the surface microstructure of flexible pressure-sensitive films is significant and highly desirable.

Herein, a novel low-cost method was developed for pressure-sensitive materials without using any molds. Polydimethylsiloxane (PDMS)/MXene/single-walled CNT (SWCNT) microspheres were obtained by using a high-pressure extrusion system and electrostatic self-assembly (ESA) technology. The conductive silica gel films with pitted microstructure were obtained using a three-dimensional (3D) printed mold as flexible electrodes. The resulting sensors exhibited a high sensitivity of 2.6 kPa^−1^, a response time of 56 ms, and a detection limit of 5.1 Pa, combined with good cyclic stability and time stability. The preparation method and sensing mechanism of piezoresistive structures of conductive elastic microspheres were examined, and feasibility was demonstrated through human health monitoring. The fabrication method of conductive elastic microspheres and electrodes proposed in this study provides a new approach to improving the performance of pressure sensors.

## 2. Experimental Section

### 2.1. Materials

The MXene-single layer Ti_3_C_2_ dispersion (5 mg·mL^−1^) was purchased from Zhengzhou Feynman Technology Co., Ltd. (Zhengzhou, China). SWCNTs (95%) material was purchased from Shanghai McLean Biochemical Technology Co., Ltd. (Shanghai, China). Sodium lauryl sulfate (98.5%) was purchased from Biofroxx (Pfungstadt, Germany). Dimethyl silicone oil, chitosan (95%), and hydrochloric acid were purchased from Aladdin Reagent Co., Ltd., Shanghai (Shanghai, China). Sylgard 184, which is composed of PDMS A (base, consisting of vinyl-terminated siloxane oligomers) and PDMS B (curing agent, consisting of siloxane oligomers and catalyst), was purchased from Dow Corning, USA (Midland, MI, USA). Conductive silica gel (RG-067) was purchased from Reeco (Dongguan) Industrial Co., Ltd. (Dongguan, China). Ethanol was purchased from Sinopharm Group Chemical Reagent Co., Ltd. (Shanghai, China).

### 2.2. Fabrication of Elastic Microspheres

PDMS A, PDMS B, and dimethyl silicone oil were mixed at a mass ratio of 10:4:3 and degassed for 15 min to remove air bubbles in a vacuum oven to obtain PDMS diluted stock. A glass needle tube and a plastic syringe were connected using an AB adhesive and cured for 24 h. Then, the PDMS diluted liquid was extruded using a glass needle tube with an adjustable high-pressure extrusion system consisting of a gas cylinder and a CKD precision pressure regulator (PRE1000-8-07). The outer diameter of the glass tube was 1.00 mm, the inner diameter was 0.60 mm, the angle was 45°, and the tip openings were 40, 30, and 25 µm, respectively. A glass beaker containing an ethanol solution of sodium lauryl sulfate (5 mg·mL^−1^) was placed under the syringe to collect the droplets. Then, the glass beaker containing PDMS droplets was placed in an electric blast drying oven for curing at 60 °C for 1 h, which yielded PDMS microspheres.

### 2.3. Preparation of Conductive Microspheres

The chitosan solution (0.4 mg·mL^−1^) was added dropwise to the PDMS microspheres until the solution completely coated the microspheres and then heated and dried in an electric blast oven at 60 °C for 10 min to obtain positively charged PDMS microspheres. Aqueous solutions of MXene (5 mg·mL^−1^) with different volumes (5, 10, 15 mL) were mixed uniformly with SWCNT (0.1 g), respectively. The MXene–SWCNT mixtures of different proportions were then, respectively, added dropwise to the chitosan-modified PDMS microspheres to obtain microspheres completely coated with the mixture, and dried at 60 °C for 20 min. The process was repeated several times. To evaluate the influence of SWCNT in constructing conductive pathway, the PDMS/MXene microspheres were prepared by a similar preparation method except that the aqueous solution only contains MXene (5 mg·mL^−1^). After these treatments, the conductive microsphere-based pressure-sensitive material was obtained.

### 2.4. Preparation of Electrodes and Pressure Sensors

The hemispherical template was prepared by 3D printing technology. The liquid conductive silica gel was obtained by mixing the conductive silica gel, diluent, and curing agent in the mass ratio of 10:9:0.75. After the uniform mixing of the contents and removal of bubbles, the as-obtained mixture was poured into a 3D printed mold and cured at 80 °C in an electric blast drying oven for 25 min to obtain a conductive silica-gel-film-based flexible electrode of size 10 mm × 10 mm × 0.275 mm with pitted microstructures (R = 0.5 mm, d = 0.167 mm) which were separated by a distance of 1.8 mm. PDMS/MXene/SWCNT microspheres coated by ESA technology were used as flexible pressure-sensitive materials, and conductive silica gel films were used as upper and lower electrodes of resistive flexible pressure sensors. The nine conductive microspheres were fixed in the concave side of the conductive silica gel film, and the upper and lower electrodes of the sensor were superimposed on each other by double-sided tape attached to the edge of the film to complete the final assembly of the sensor. The discussion of parameters, such as those between conductive microspheres, is in the Supplementary Materials.

### 2.5. Characterization and Measurements

Instron universal experimental machine (5943), a current source (6221, Keithley Instruments Inc., Cleveland, OH, USA), and a nanovoltmeter (34420A, Agilent technologies Inc., Santa Clara, CA, USA) were used to test the electro–mechanical properties of the sensors as shown in Figure S4. Scanning electron microscopy (SEM, Helios-G4-CX, and S-4800) was used to characterize the microspheres. The initial resistance of PDMS microspheres was measured using a semiconductor parameter tester (4200SCS/F, Keithley). The flexible electrode material was analyzed using a dual electrical measurement four-probe tester (RTS-9).

## 3. Results and Discussion

### 3.1. Fabrication and Characterization

**Figure 1:**
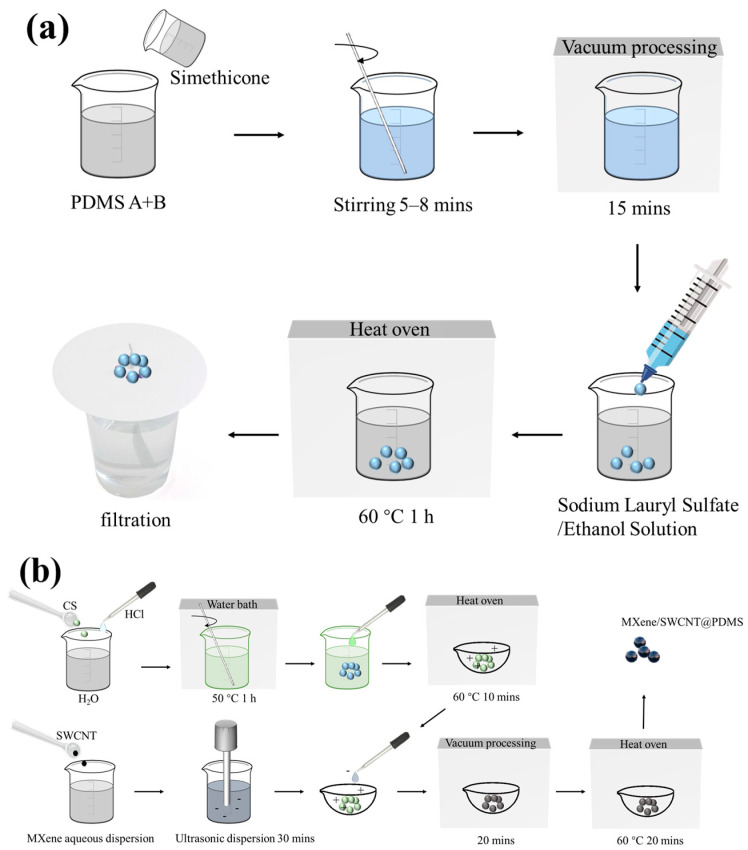
Preparation process. (**a**) PDMS microspheres; (**b**) PDMS/MXene/SWCNT microspheres.

**Figure 2:**
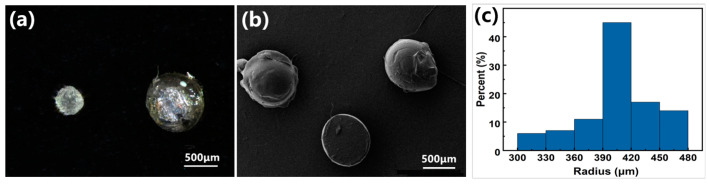
Morphological characterization of PDMS microsphere. (**a**) Optical microscopy image; (**b**) SEM image; (**c**) particle size analysis of the PDMS microsphere.

The preparation methods of PDMS elastic microspheres and conductive elastic microspheres are shown in Figure 1a and Figure 1b, respectively. The experimental group with needle tips of 25 µm diameter could not steadily output droplets within the safe working range of the pressure regulator. Experimental groups with needle tips of 40 µm and 30 µm began to slowly output droplets at about 0.8 MPa and reached saturation at 0.9 MPa (2.5 drops·min^−1^). After drying and curing, the cured PDMS microspheres were separated with filter paper. Optical microscopy was used to visually examine the PDMS microspheres, as shown in Figure 2, and the results revealed that the PDMS microspheres prepared with a combined syringe with needle tips of 40 µm consisted of a large diameter (close to 1 mm in diameter, right image in Figure 2a) and a rough surface. The PDMS microspheres prepared with a 30 µm combination syringe were characterized by SEM. Figure 2b shows that the surface of the PDMS microspheres was smooth. The particle size uniformity analysis was performed on 100 PDMS microspheres prepared with a 30 µm combination syringe. Figure 2c exhibits that the radius of the small spheres is distributed from 300 to 480 µm, and the homogeneity is high, which is of great significance for the construction of the surface microstructure of flexible pressure-sensitive materials. Accordingly, the optimal tip opening diameter of the glass needle was selected as 30 µm for subsequent experiments.

After the PDMS elastic microspheres were successfully prepared, the PDMS elastic microspheres were modified by the ESA process. The basic principle of ESA technology involves the use of electrostatic attraction to assemble two or more ionic molecules into multilayer films on the surface. This strategy can be used to prepare high-quality and large-area materials. Sensors obtained by this method access advantages such as fast response, high sensitivity, and low cost. Figure 1b illustrates that PDMS microspheres were first modified with positively charged chitosan, and then MXene-SWCNT film was coated on PDMS microspheres by the ESA method. The preparation route of PDMS/MXene microspheres was similar to that of PDMS/MXene/SWCNT microspheres.

The macroscopic differences between PDMS/MXene/SWCNT microspheres obtained by the ESA method and pure PDMS microspheres are shown in Figure S1. Pure PDMS microspheres are transparent, and the coating of MXene-SWCNT film makes the microspheres black. Compared with the PDMS/MXene microspheres directly coated with MXene films by vacuum-assisted layer-by-layer self-assembly method, the PDMS/MXene microspheres coated by the ESA method showed a uniform distribution of MXene layers on the surface. The MXene films presented a multi-layer structure (Figure S1b,c). The surface uniformity and conductive layer coverage of PDMS/MXene/SWCNT microspheres obtained by the ESA method were much higher than those of PDMS/MXene/SWCNT microspheres directly coated by vacuum-assisted layer-by-layer self-assembly method (Figure S1d,e). Figure S1g shows the PDMS microspheres directly coated with SWCNT, proving that it is not SWCNT that leads to the uniform distribution of conductive layer on the surface of the PDMS microspheres. Figure S1f demonstrates that the surface morphology of PDMS/MXene/SWCNT microspheres after two ESA coating cycles is similar to that of PDMS/MXene/SWCNT microspheres after one-time coating. Figure S1h shows PDMS/MXene/SWCNT microspheres that have undergone three ESA coating cycles, indicating that excessive coating times may have a negative effect on the uniformity of the conductive layer distribution on the surface of the PDMS microsphere. In summary, MXene and SWCNT can be successfully coated on the surface of PDMS microspheres through ESA.

**Figure 3:**
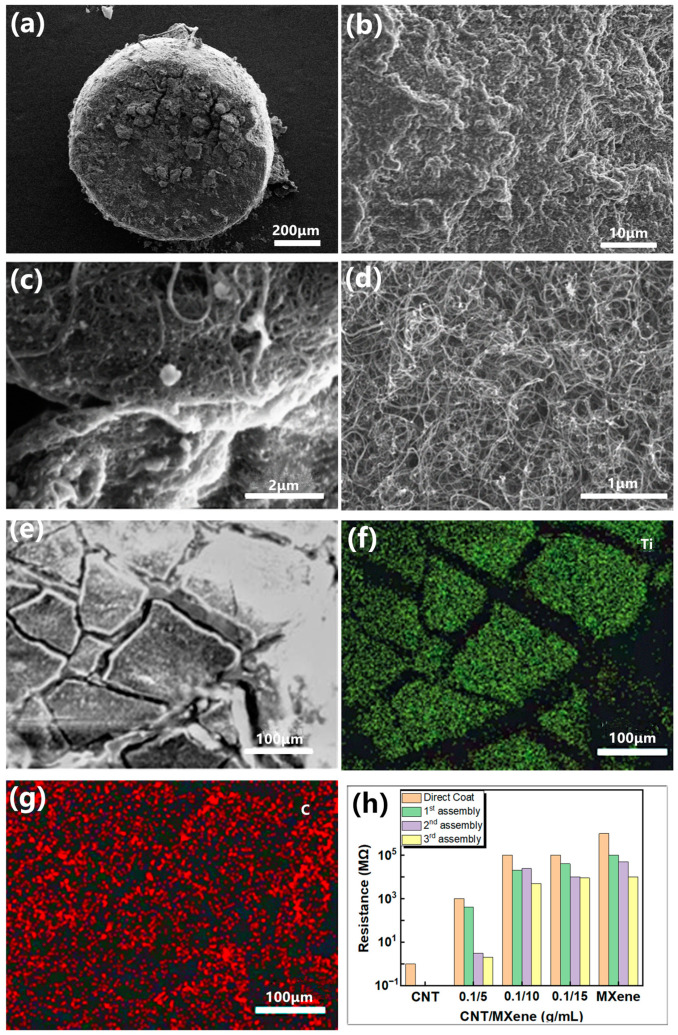
SEM and EDX characterization of PDMS/MXene/SWCNT microspheres and initial resistance. (**a**–**e**) SEM image of PDMS/MXene/SWCNT (ESA-2) microspheres; (**f**,**g**) EDX characterization of the distribution of Ti and C elements on the surface of the microsphere; (**h**) initial resistance of PDMS microspheres modified by different methods.

The surface morphologies of the microspheres obtained by ESA were analyzed by SEM. Figure 3a–d illustrate that MXene–SWCNT revealed a stacked layer-on-layer assembly evenly distributed on the surface of PDMS microspheres. Further, the enlarged SEM images show that SWCNTs intertwine with each other to form a mesh interweaving structure on the MXene lamellae, and they together form a 3D structure, which can increase the interlayer spacing of MXene. This void structure can provide a larger deformation range, thereby improving the sensitivity and detection range of the MXene layer. Figure 3e–g exhibit that the Ti element is evenly distributed on each layer. Additionally, the C element shows uniform bright areas in each layer, and uniform dark areas also appear in the interlayer, indicating that SWCNT causes the correlation between MXene layers. 

Figure 3h shows the initial resistance of PDMS microspheres modified by different means. At a concentration ratio of SWCNT 0.1 g/ MXene 5 mL, the best electrical conductivity was recorded. The incorporation of SWCNT significantly reduced the initial resistance of microspheres, and the synergistic effect generated by the combination of SWCNT and MXene promoted the construction of conductive networks. The unit resistance of the PDMS/MXene/SWCNT microspheres determined through a single ESA coating was 400 MΩ. This value reduced from 400 MΩ to 3 MΩ and 2 MΩ after two and three ESA coatings, respectively. The surface film of PDMS/MXene/SWCNT microspheres obtained by the ESA coating technique was more complete and a better conductive path was formed than that of the groups without chitosan modification. Although the conductivity of the microspheres gets improved with the increase of the number of ESA coating cycles, the uniformity of the conductive layer on the surface of the PDMS microspheres may be negatively affected by more than two coating cycle times as shown in Figure S1e,f,h. The PDMS/MXene/SWCNT film obtained after two cycles of ESA coating not only shows high uniformity but also can meet the standard of constructing conductive sensor networks. Therefore, the PDMS/MXene/SWCNT microspheres obtained after two ESA coating cycles were selected for subsequent experiments.

**Figure 4:**
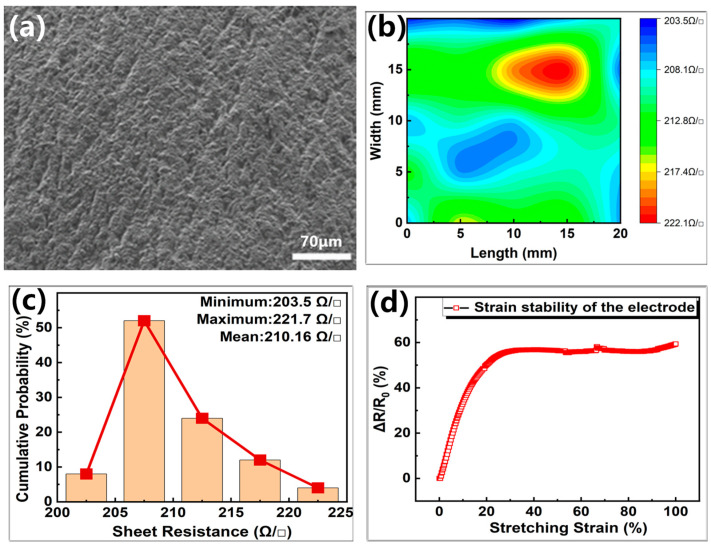
SEM image and electrical properties of the flexible electrode. (**a**) SEM image of the flexible electrode; (**b**) the mapping image of sheet resistance; (**c**) the corresponding statistical histograms calculated from (**b**); (**d**) electrical tensile stability test for standard tensile parts.

Noteworthy, the surface morphologies and electrical properties of the flexible electrodes also play an important role in sensors; therefore, SEM characterization of the conductive silica gel flexible electrodes was carried out. Figure 4a exhibits that the surface of the electrodes revealed rough structures similar to orange peel, which is beneficial for the stability of PDMS/MXene/SWCNT conductive microspheres under pressure. A conductive silica gel film of size 10 mm × 10 mm × 0.275 mm was prepared through tablet press and blade coater to study the electrical properties of electrode material. The electrical properties of the 0.275 mm thick electrode material shown in Figure 4b,c revealed a mean square resistance of the film to be 210.16 Ω/□ and radial non-uniformity of 8.56%, indicating good electrical conductivity. Nine points were uniformly selected on the film, and their thickness was measured with a thickness gauge. Figure S9 shows that the thickness difference measured is within 2 μm, which indicates relatively uniform thickness of the films and the average value obtained is 0.275 mm. The Rq roughness of the conductive silica gel films is 300 nm, and the AFM image is shown in Figure S10. The average resistivity of the 0.275 mm thick films was estimated to be 6.285 Ω·cm, with conductivity much higher than that of PDMS/MXene/SWCNT conductive microspheres, revealing the suitability of the electrode materials for sensing.

The dumbbell-shaped standard tensile part was obtained by pouring liquid silica gel at the same ratio as that of the prepared electrode into the national standard GB/T 528-2009 tensile part mold [45], followed by curing at 80 °C in the electric blast drying oven for 25 min. Figure 4d presents that the material showed good electrical tensile stability within a small tensile range.

### 3.2. Mechanism Analysis

In the present study, the resistive flexible pressure sensors converted pressure signals into changes in resistance, which can be defined by the formula: R = ρL/S, where ρ is the resistivity, L is the length, and S refers to the cross-sectional area. Notably, L and S may change with the shape and size of the material, resulting in varying resistance. Accordingly, when the external pressure was applied to the pressure sensor, it resulted in sensor deformation. For composite materials widely used in resistive flexible pressure sensor applications, the application of pressure would result in variation in the distance. Based on the infiltration theory and tunnel current effect, when the conductive filler is incorporated into the polymer substrate with high resistivity to form a composite material, the external pressure leads to a change in the distribution of the conductive filler in the composite material, and the conductive filler forms a conductive network in the composite material, leading to the decrease in its resistance. Moreover, the existence of microstructures between piezosensitive materials and the electrodes leads to the decline in the resistance of the device as a function of the contact area between the piezosensitive material and the electrodes.

**Figure 5:**
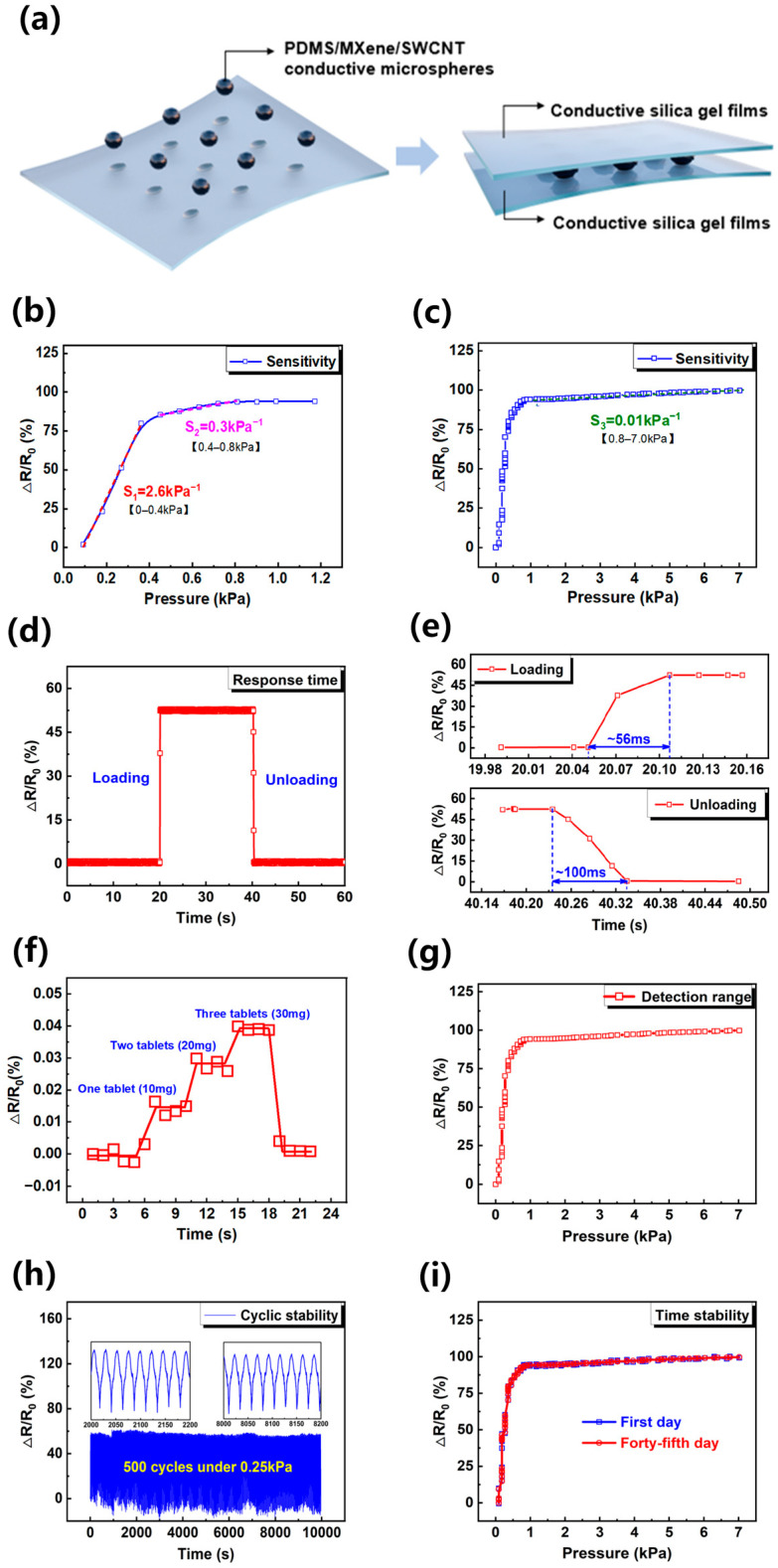
(**a**) Sensor structure diagram; (**b**) pressure response curve of the piezoresistive sensor within 0–0.8 kPa; (**c**) pressure response curve of the piezoresistive sensor within 0–7.0 kPa; (**d**) device real-time response curve; (**e**) device response time diagram and device recovery time diagram; (**f**) sensor response to weak stimuli; (**g**) sensor detection range; (**h**) the 500-cycle curve of the flexible piezoresistive sensor; (**i**) pressure response curve before and after static setting.

The pressure-sensitive material and microstructure design of the resistive pressure sensor played a key role in the sensing performance. Figure 5a shows that the microsphere-structured pressure-sensitive material was sandwiched between the conductive silica gel electrodes. Under the action of ESA, MXene/SWCNT film yielded many clusters, forming two types of potential conductive paths. The first conductive path is formed by surface contact between adjacent MXene/SWCNT clusters as shown in Figure S2 at low pressure, and this conductive path rapidly saturates with the increase of pressure, which also leads to the highest sensitivity of the sensor (2.6 kPa^−1^) in the range of 0–0.4 kPa. The other conductive path is formed when the contact points between SWCNT increase or even become linear contact (shown in Figure S3) due to the compression deformation of the material in the medium pressure range. The formation of this conductive path makes the sensor have a sensitivity of 0.3 kPa^−1^ over a pressure range of 0.4–0.8 kPa. Moreover, the contact area between the MXene/SWCNT film with the upper and lower electrodes increased as a function of external pressure, resulting in further reduction of the resistance in a wide pressure range. With the continuous increase in the pressure, the increasing rate of the contact area between the MXene/SWCNT film and the electrodes started to diminish, leading to the saturated sensitivity of the pressure sensor. This feature makes the sensor have a sensitivity of 0.01 kPa^−1^ over a pressure range of 0.8–7 kPa.

### 3.3. Sensing Performance

The sensing performance of the pressure sensor was evaluated, and the results are shown in Figure 5b–i. In general, the sensitivity of the pressure sensor S can be defined by the formula: S = (ΔR/R_0_)/ΔP, where R_0_ is the initial resistance, ΔR is the change in resistance, and P is the applied pressure. For these experiments, the sensor was first placed in the Instron universal experimental machine (5943), and constant pressure was then applied to the sensor through the 10 mm × 10 mm upper indenter as shown in Figure S6. The corresponding real-time pressure information is recorded in the main machine of the universal testing machine (5943). The sensors were measured at 25 °C and 45% relative humidity. In the test, the current source (6221, Keithley) provided a constant current of 0.06 mA, and the initial output voltage was 8.4 V. The real-time changes in resistance were obtained by processing the current and voltage data obtained from the current source (6221, Keithley) and nanovoltmeter (34420A, Agilent). The electrical response with the loading pressure could be used as a standard reference to the sensor. Figure 5b,c illustrate that the proposed sensor displayed three different sensitivity stages. At applied pressure below 0.4 kPa, the sensitivity of the PDMS/MXene/SWCNT microsphere-based resistive pressure sensor reached 2.6 kPa^−1^, a value 173 times than that of the resistive pressure sensor based on pure Mxene manufactured by ESA technique (0.015 kPa^−1^) [46]. At a pressure of 0.4–0.8 kPa, the sensitivity of the PDMS/MXene/SWCNT microspheres-based resistive pressure sensor was estimated to be 0.3 kPa^−1^. With the continuous increase in the pressure, the sensitivity of the pressure sensor declined to 0.01 kPa^−1^. Therefore, the PDMS/MXene/SWCNT microspheres-based resistive pressure sensor possessed a high sensitivity over a wide pressure range. In addition, the electrical response with the loading pressure of six sensors was tested to verify the reproducibility of the device as shown in Figure S5. The results showed that their electrical response to pressure is highly consistent. 

The real-time response ability of the PDMS/MXene/SWCNT microspheres-based resistive pressure sensor was measured with the Instron universal experimental machine under an applied pressure of 0.27 kPa to the sensor. The time taken for the sensor resistance to fall from the initial value to the corresponding resistance value under the pressure was defined as the loading response time of the sensor. Figure 5d,e depict that the loading response time of the sensor was about 56 ms. After releasing the pressure, the unloading response time of the sensor reached 100 ms. Thus, the pressure loading/unloading experiments of PDMS/MXene/SWCNT microsphere-based resistive pressure sensor revealed a real-time response ability, which shows practical significance for the development of wearable dynamic monitoring equipment.

The ability of the PDMS/MXene/SWCNT microspheres-based resistive pressure sensor to detect weak signals was evaluated by placing a vitamin tablet with a mass of 50 mg repeatedly on the sensor. Figure 5f demonstrates that when the tablet was placed (about 5.1 Pa), the sensor produced a distinct and identifiable response. Moreover, the sensor output signal was relatively stable upon stacking three tablets upright (about 15.3 Pa). The detection range of the sensor tested in Figure 5g reveals a sensor working normally in the range of 0–7 kPa.

Figure 5h shows that the sensor was tested for 500 loading/unloading cycles under 0.25 kPa, and the results indicated that it maintained a relatively stable signal output. After 500 long cycles (shown in Figure S11), the surface of PDMS/MXene/SWCNT microspheres did not change much from the initial morphology. The MXene-SWCNT film was still completely coated on the surfaces of the microspheres. The application environment of the sensor in the life scene was simulated by performing air stability tests of the resistive pressure sensor based on PDMS/MXene/SWCNT microspheres. Figure 5i exhibits that the force–electric performance curve output of the sensor after 45 days of standing in the air illustrated almost no difference from the initial one, which proved that the sensor had good stability. The sensor also exhibited good sustainability (shown in Figure S12). Overall, the proposed sensor showed good comprehensive performance in terms of high sensitivity, wide detection range, low detection limit, and good stability.

### 3.4. Practical Applications

**Figure 6:**
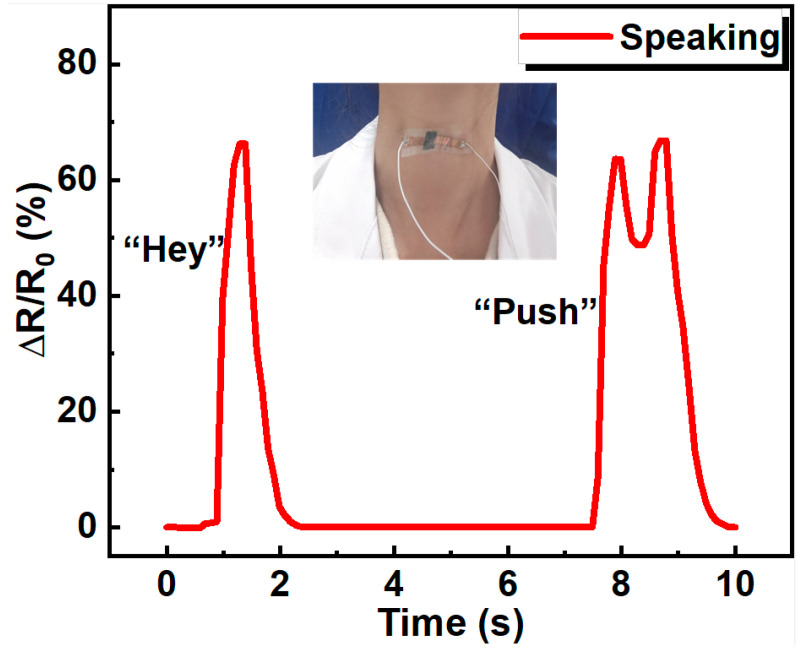
The application of the piezoresistive sensor in word pronunciation differentiation.

Because of the high sensitivity and good stability, the resistive pressure sensor based on PDMS/MXene/SWCNT microspheres can be used in human health monitoring. With conductive silica gel film flexible electrodes, the prepared PDMS/MXene/SWCNT microsphere-based flexible pressure sensor is thin and lightweight. The sensor can be easily attached to different places of the human body with the help of polyurethane (PU) medical tape. As shown in Figure 6, the device was attached to the throat skin to capture the vocal cord vibration during speaking. when the subject pronounced the word “Hey”, the sensor showed one characteristic peak. When the subject pronounced the word “Push”, the sensor showed two characteristic peaks, corresponding to “Pu” and “sh”, proving the pressure sensor’s ability to distinguish the pronunciation of different words.

**Figure 7:**
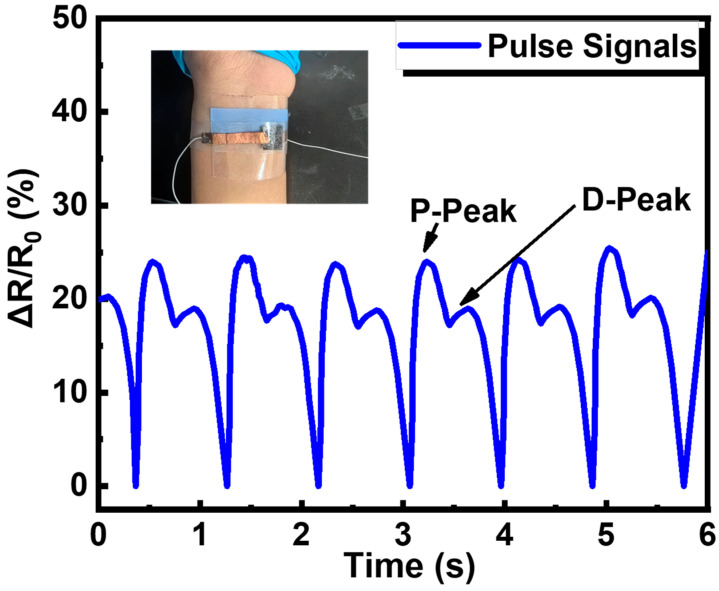
The application of the piezoresistive sensor in human pulse signals monitoring.

Besides, the piezoresistive sensor was placed on the radial artery of an adult female’s wrist to measure pulse (Figure 7). The pulse rate was measured as 67 beats per minute, and the two characteristic peaks (percussion peak and diastolic peak) of the radial pulse were recorded, demonstrating the pressure sensor’s ability to detect human pulse signals. These results indicate that the resistive pressure sensor based on PDMS/MXene/SWCNT microspheres has a good application prospect in the field of human health monitoring.

## 4. Conclusions

This study developed a sensitive piezoresistive sensor based on PDMS/MXene/SWCNT microspheres, with excellent sensing performance, by a simple and facile production process. The conductive silica gel film with pitted microstructure was obtained by using the 3D printed mold, as the upper and lower electrodes. The formation of various conductive paths in the pressure-sensitive medium resulted in significant changes in resistance under pressure. The optimized sensor exhibited a high sensitivity of 2.6 kPa^−1^, response time of 56 ms, detection limit of 5.1 Pa, and good cyclic stability and time stability. Thus, the sensor shows promising potential for broad human health monitoring. Besides, the fabrication method of conductive elastic microspheres and electrodes can be extended for the fabrication of sensors for various wearable electronic devices.

## Data Availability

Data are contained within the article and Supplementary Materials.

## Supplementary Materials

The supporting information can be downloaded at https://www.mdpi.com/article/10.3390/s24051640/s1. Reference [47] is cited in Supplementary Materials.
