# Supplementary material for: Highly Sensitive Pressure Sensor Based on Elastic Conductive Microspheres
Source: Sensors (Basel). 2024 Mar 2;24(5):1640. doi: 10.3390/s24051640 (PMC10934857; doi:10.3390/s24051640)
Supplement: Supplementary file 1 [file sensors-24-01640-s001.zip › sensors-2823604-supplementary.pdf]

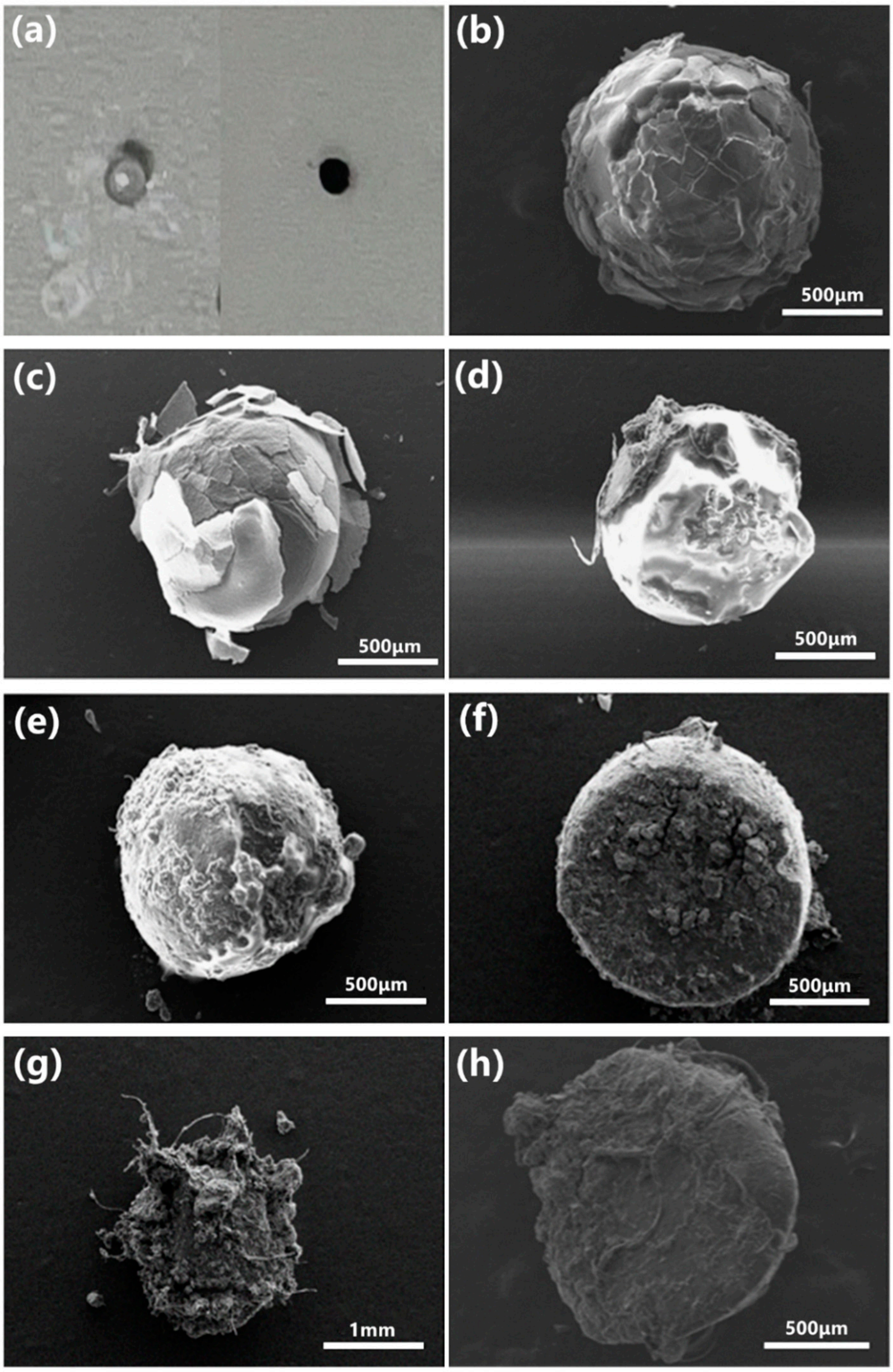

Figure S1. Morphology characterization of conductive microspheres. (a) Pure PDMS microspheres and PDMS/MXene/SWCNT(ESA-1) microspheres. (b) SEM images of PDMS/MXene(ESA-1) microspheres. (c) SEM images of PDMS/MXene (Non-ESA) microspheres. (d) PDMS/MXene/SWCNT(Non-ESA) microsphere SEM. (e) PDMS/MXene/SWCNT(ESA-1) microsphere SEM. (f) PDMS/MXene/SWCNT(ESA-2) microsphere SEM. (g) SEM image of PDMS/SWCNT(Non-ESA) microsphere. (h) SEM image of PDMS/MXene/SWCNT(ESA-3) microsphere.

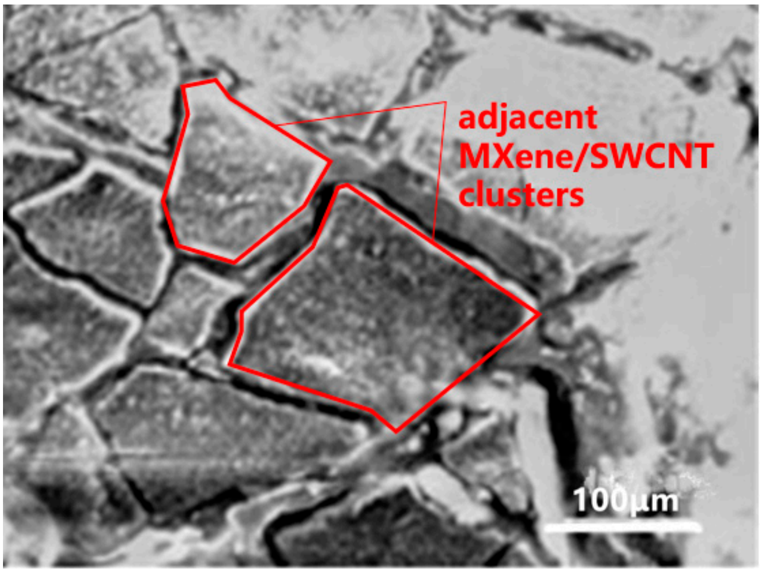

Figure S2. Adjacent MXene/SWCNT clusters in SEM image of PDMS/MXene/SWCNT(ESA-2) microspheres.

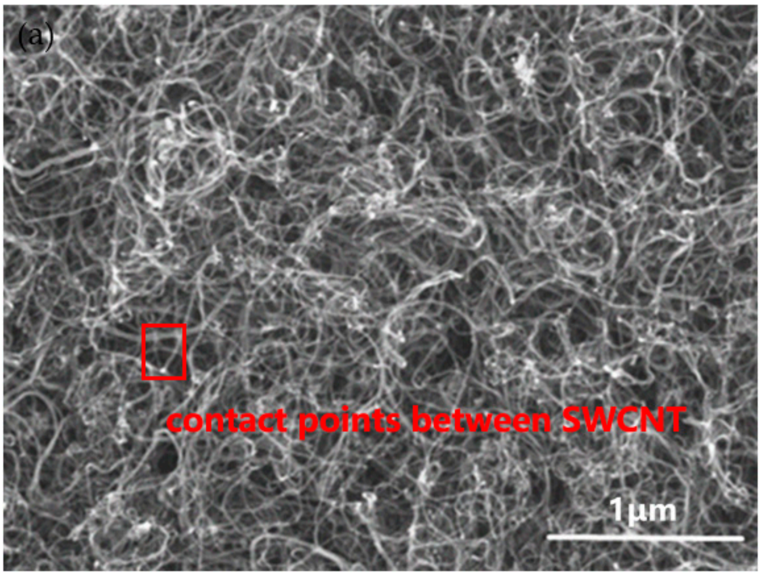

Figure S3. Contact points between SWCNT in SEM image of PDMS/MXene/SWCNT(ESA-2) microspheres.

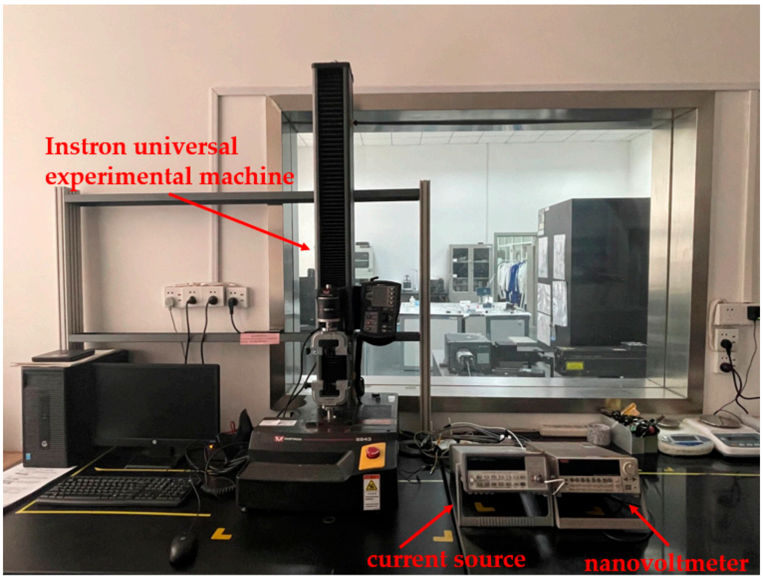

Figure S4. Electrokinetic analysis system.

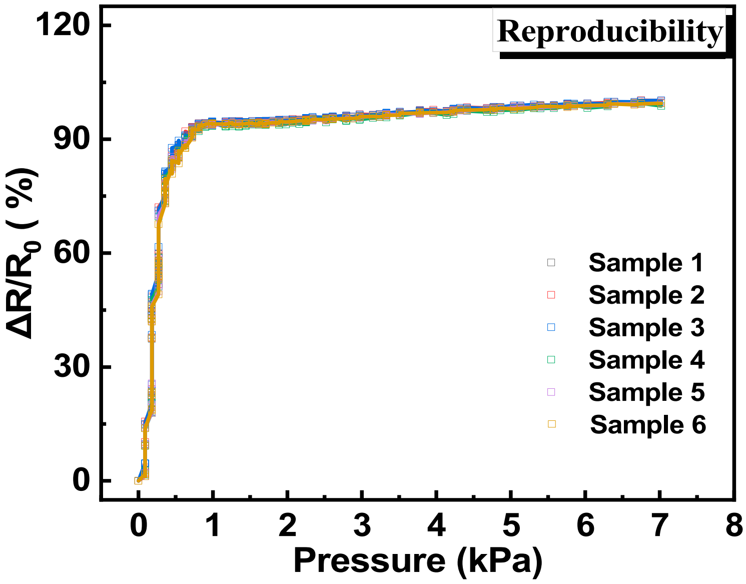

Figure S5. Relative electrical response of piezoresistive sensor observed on six samples prepared from same batch as a function of the loading pressure.

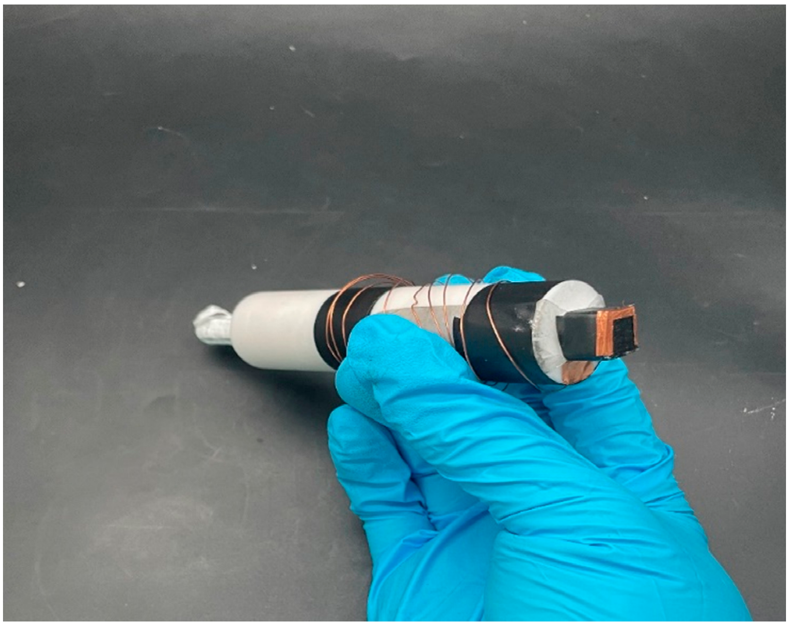

Figure S6. Upper indenter.

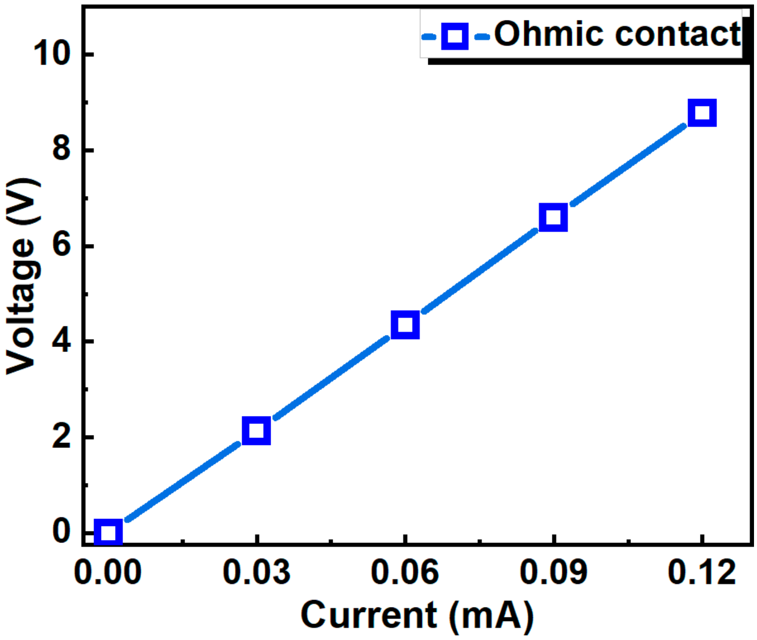

Figure S7. I-V behavior of the sensor under an applied pressure of 0.27 kPa.

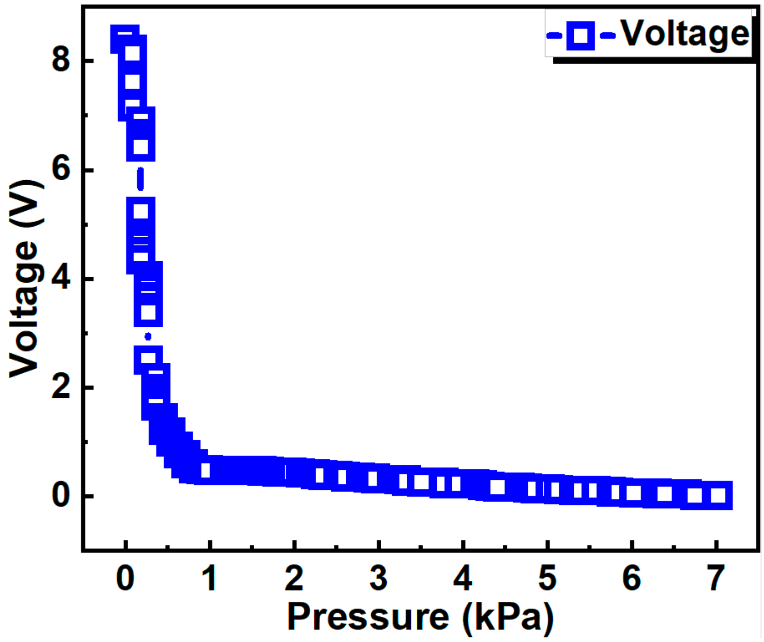

Figure S8. The output voltage of the sensor under different pressures.

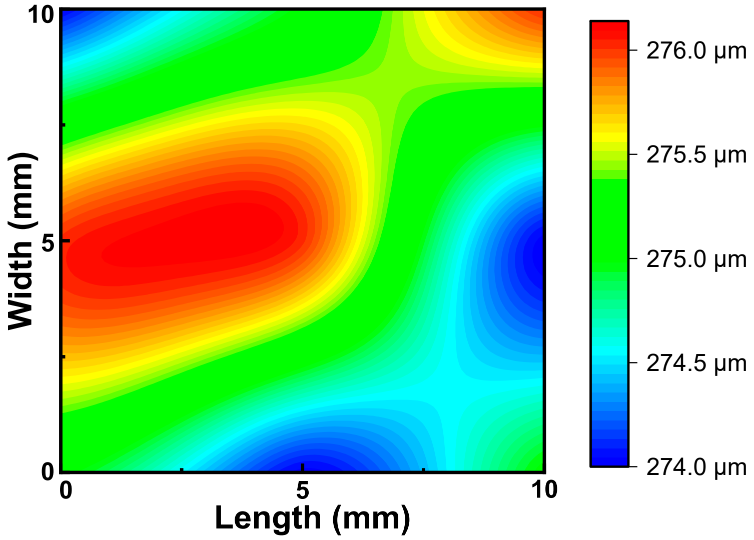

Figure S9. The mapping image of film thickness.

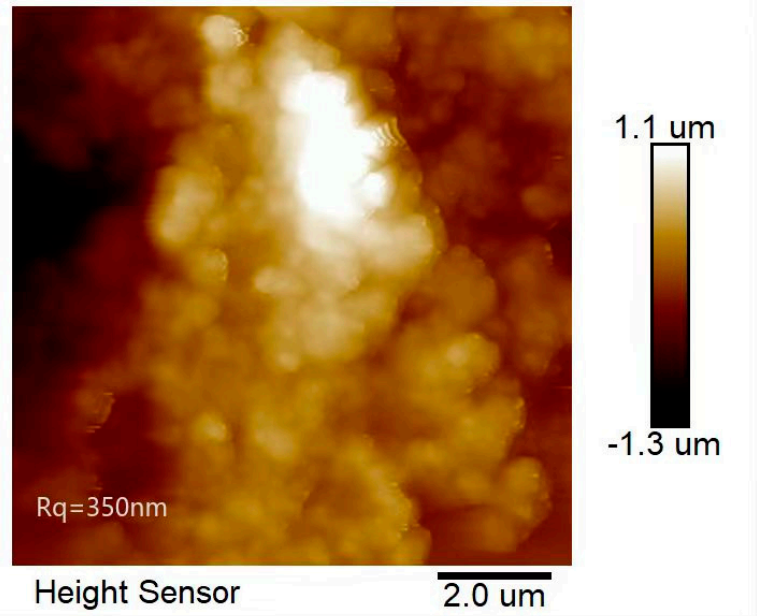

Figure S10. AFM image of conductive silica gel film with Rq roughness of 350 nm.

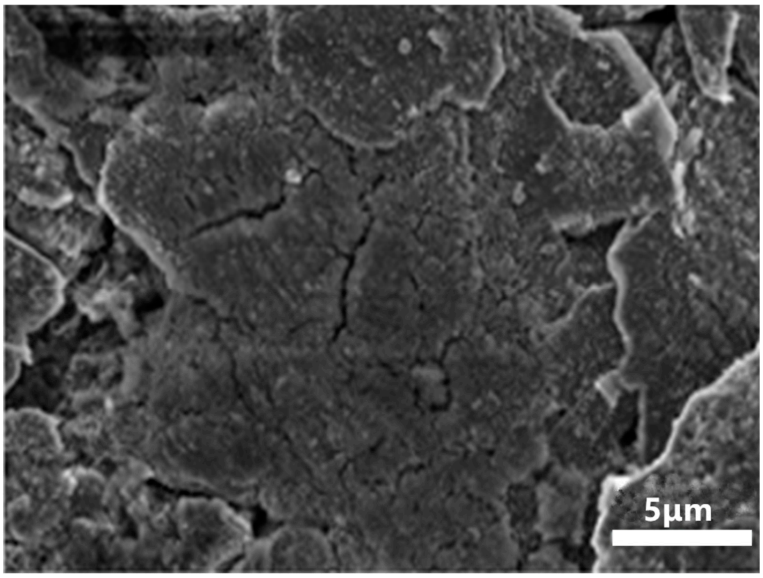

Figure S11. SEM image of surface of PDMS/MXene/SWCNT microsphere after 500-cycle curve.

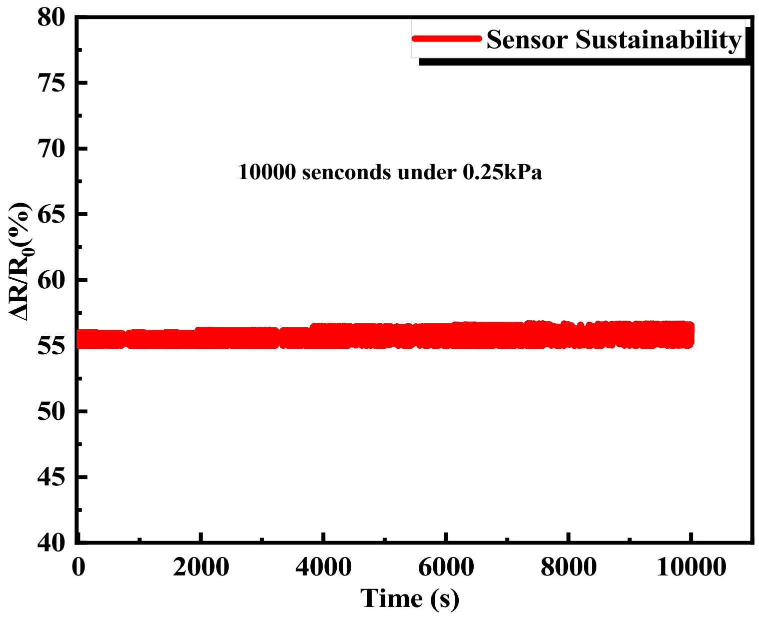

Figure S12. Variation of the resistence values at the external pressure's value of 0.25kPa for 10000 seconds.

Discussion of electrodes parameters

In order to make the sensor have good sensing performance, the sensor should have the largest microstructure size and the smallest spacing [1]. In the experiment, the microsphere size is fixed. By trying to prepare flexible electrodes with different microsphere spacing, it was found that 1.8mm is the electrode with the smallest microsphere spacing that can be obtained under the current process. And with this spacing, 9 conductive microspheres were placed in the sensor to make the resistance within the measuring range of the electrical stations.

Confirmation of PDMS/MXene/SWCNT structures

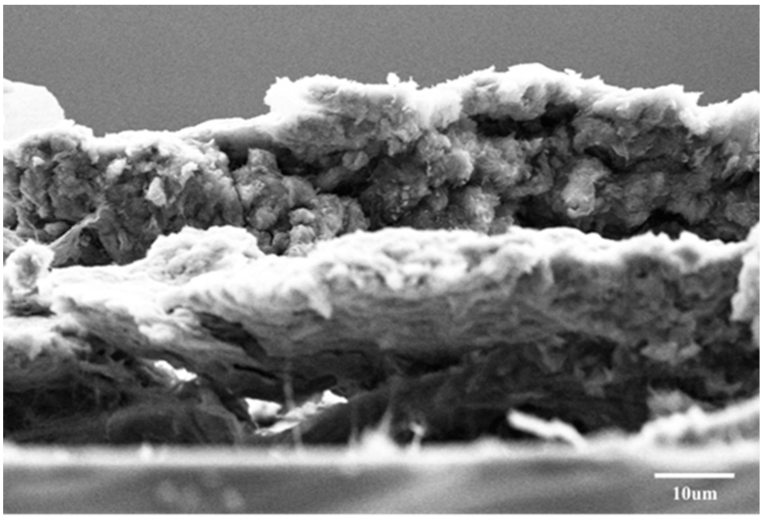

Figure S13. SEM image of cross-section of quenched film of PDMS/MXene/SWCNT (ESA-2).

To analyze the structure and components formed on the surface of the conductive microsphere, PDMS/MXene/SWCNT film was prepared by the same method after two electrostatic self-assembly, and was quenched with liquid nitrogen. Then the cross-section of quenched film was observed by SEM. Figure S13 shows that MXene layers are stacked on the surface of PDMS, and the gaps formed by SWCNT are in the middle.

Deformation limitation of the surface of the PDMS/MXene/SWCNT microspheres

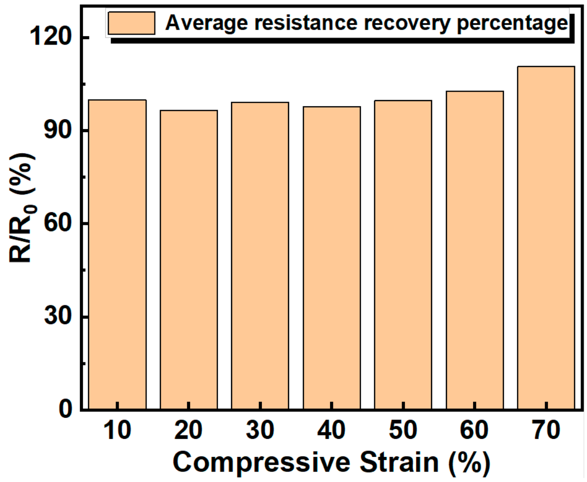

Figure S14. average resistance recovery percentage of the conductive microsphere after experiencing different degrees of strain were tested.

To analyze the deformation limit of the surface of the *PDMS/MXene/SWCNT* microspheres, the resistance recovery of each group of 5 conductive microspheres prepared from same batch used in the sensor after experiencing different degrees of strain were tested. Figure S14 showed that the average resistance recovery percentage of the conductive microsphere exceeded 110% after 70% deformation, while the resistance value recovered about 100% when the deformation was below 70%, indicating that the maximum deformation limit of the conductive microsphere surface can hold is about 60% , and at 70% compressive strain, the conductive path on the surface of microsphere is irreversibly obviously destroyed.

The application of the piezoresistive sensor in acupoint pressure detection

In traditional Chinese medicine, meridians diagnosis and treatment are used for the treatment of diseases, in which acupoint massage stimulates specific parts of the human body through pressure. This method has been widely used in clinical diagnosis and treatment, since it can dredge meridians, promote blood circulation, and regulate diseases. However, the stimulation intensity and time required by different acupoints vary significantly, and the complexity of acupoint massage and its therapeutic effect relies on the doctor’s medical experience and massage techniques. In this study, the intensity and duration of pressure stimulation were evaluated during acupressure to provide data basis for customizing appropriate massage acupoint pressure for users' physical conditions by massage instruments.

The sensors were calibrated to obtain the standard reference through the Instron universal experimental machine (5943) and electrical stations in the same way as in 3.3. After calibration,

the sensors were connected to the current source and the nanovoltmeter which connected with a computer on subjects' points at 25℃ and 45% relative humidity. Figure S15 shows that the sensor was attached to several points by medical application: Neiguan point (volar side of the arm from the second finger of the horizontal stripe of the wrist), Hegu point (the middle depression of the second metacarpal bone), and Zhongwan point (upper abdomen, anterior midline of the human body, four fingers above the navel). Professional doctors then stimulated the three points with different intensities and modes. The corresponding real-time electrical response was recorded in the computer and analyzed. The results confirmed that the sensor successfully detected pressure signals under different modes. The pressure of the Neiguan point was applied with a small force, slow pressure, and slow release. The data obtained from the sensor under this pressure suggested a uniform increase during the application stage, reaching the maximum pressure of 0.21 kPa after about 247 ms, followed by a uniform decline in the process of force discharge for about 203 ms.

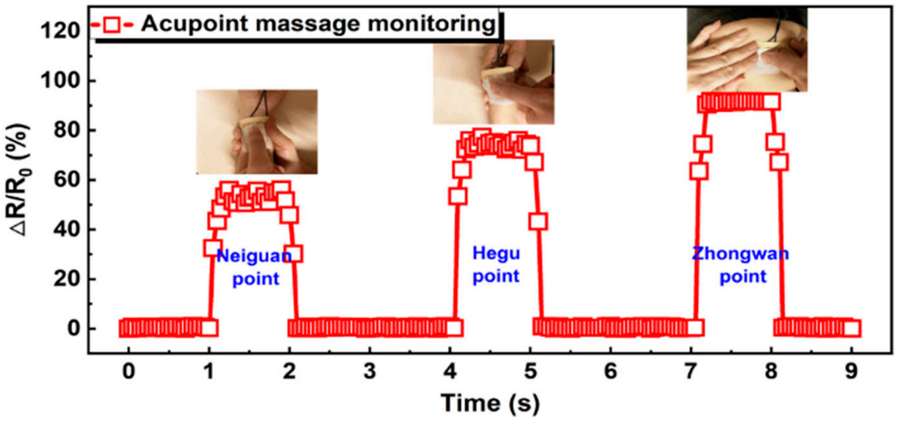

Figure S15. The application of the piezoresistive sensor in acupoint pressure detection.

For pressing Hegu point, the method of moderate force, slow pressure, and quick release was adopted. The data displayed a uniform increase in the force by the sensor during the pressing stage, reaching a maximum pressure of 0.30 kPa after about 200 ms. During the unloading process, the force decreased uniformly, and the time was about 152 ms.

The pressure at Zhongwan point was applied with larger forces, first at slow rate and then at fast rate, which was followed by fast release before slow release. The results revealed an applied force reaching 0.24 kPa after about 84 ms, and 0.74 kPa after 202 ms. During force discharge, the applied force decreased to 0.28 kPa after about 56 ms and the unloading was completed after about 155 ms. Since the sensor prepared by this method is simple and suitable for large-scale production, it may provide a hardware basis for a large number of data acquisition. The appropriate pressing mode of different acupoints can be obtained by collecting and analyzing the large data of acupressure for relevant practitioners, increasing the learning efficiency of relevant massage techniques.

Reference

1. Zhang, Y.; Han, F.; Hu, Y.G.; Xiong, Y.X.; Gu, H.; Zhang, G.Q.; Zhu, P.L.; Sun, R.; Wong, C.P. Flexible and Highly Sensitive Pressure Sensors with Surface Discrete Microdomes Made from Self-Assembled Polymer Microspheres Array. *Macromol. Chem. Phys.* **2020**, *221, 2000073.*
